# Supplementary material for: SenSet, a novel human lung senescence cell gene signature, identifies cell-specific senescence mechanisms
Source: bioRxiv. 2024 Dec 22:2024.12.21.629928. Preprint. [Version 1] doi: 10.1101/2024.12.21.629928 (PMC12279080; doi:10.1101/2024.12.21.629928)
Supplement: Supplement 1 [file media-1.pdf]

Supplement: SenSet, a novel human lung senescence cell gene signature, identifies cell-specific senescence mechanisms

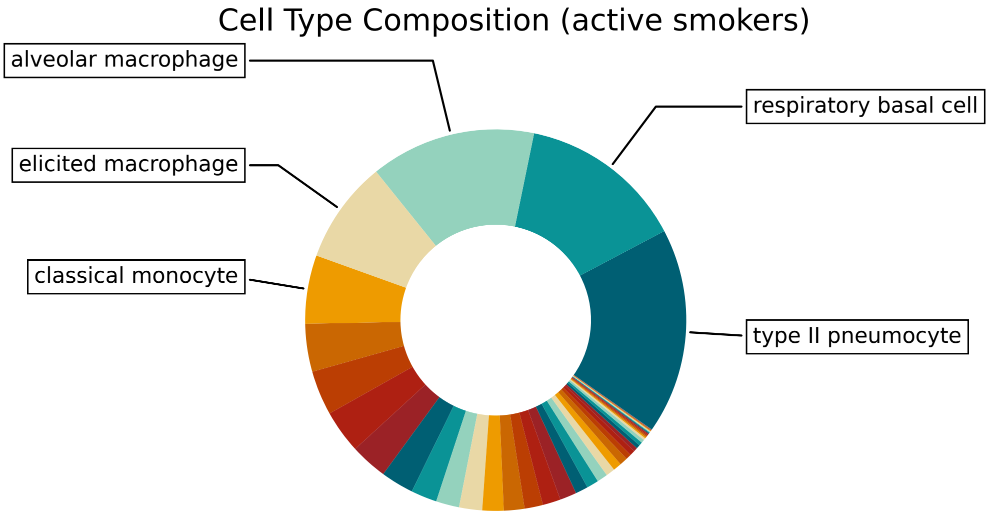

Figure 1: **Cell type proportion among active smokers.**

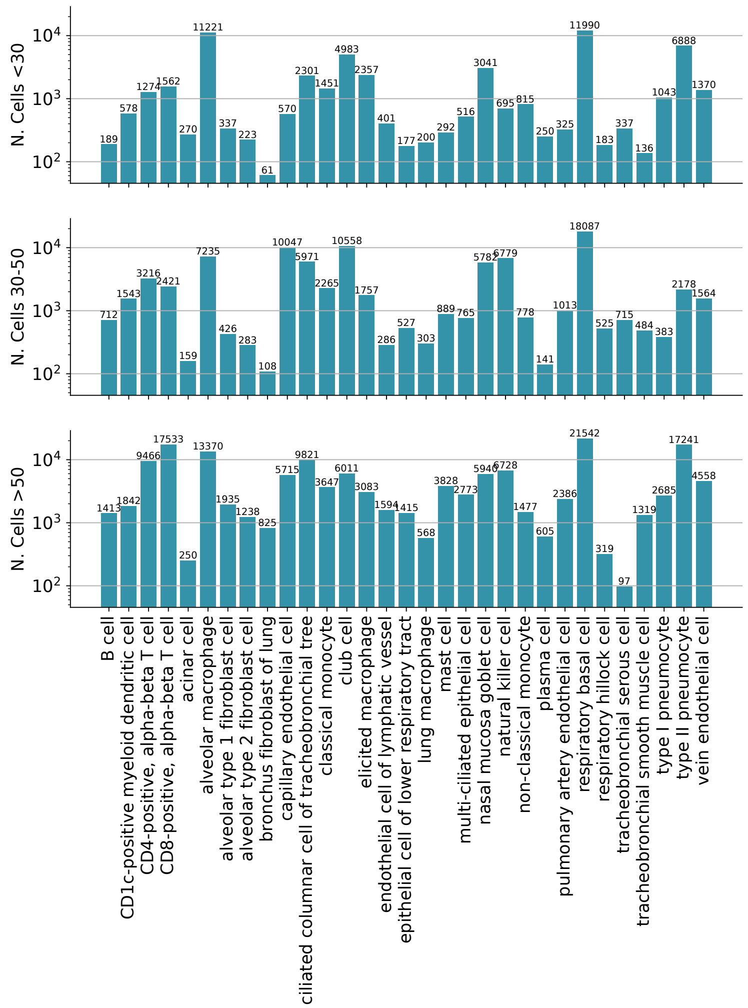

Figure 2: **Number of cells for each cell type used for PU learning and each age group in the HLCA dataset. Data from non-smokers only.**

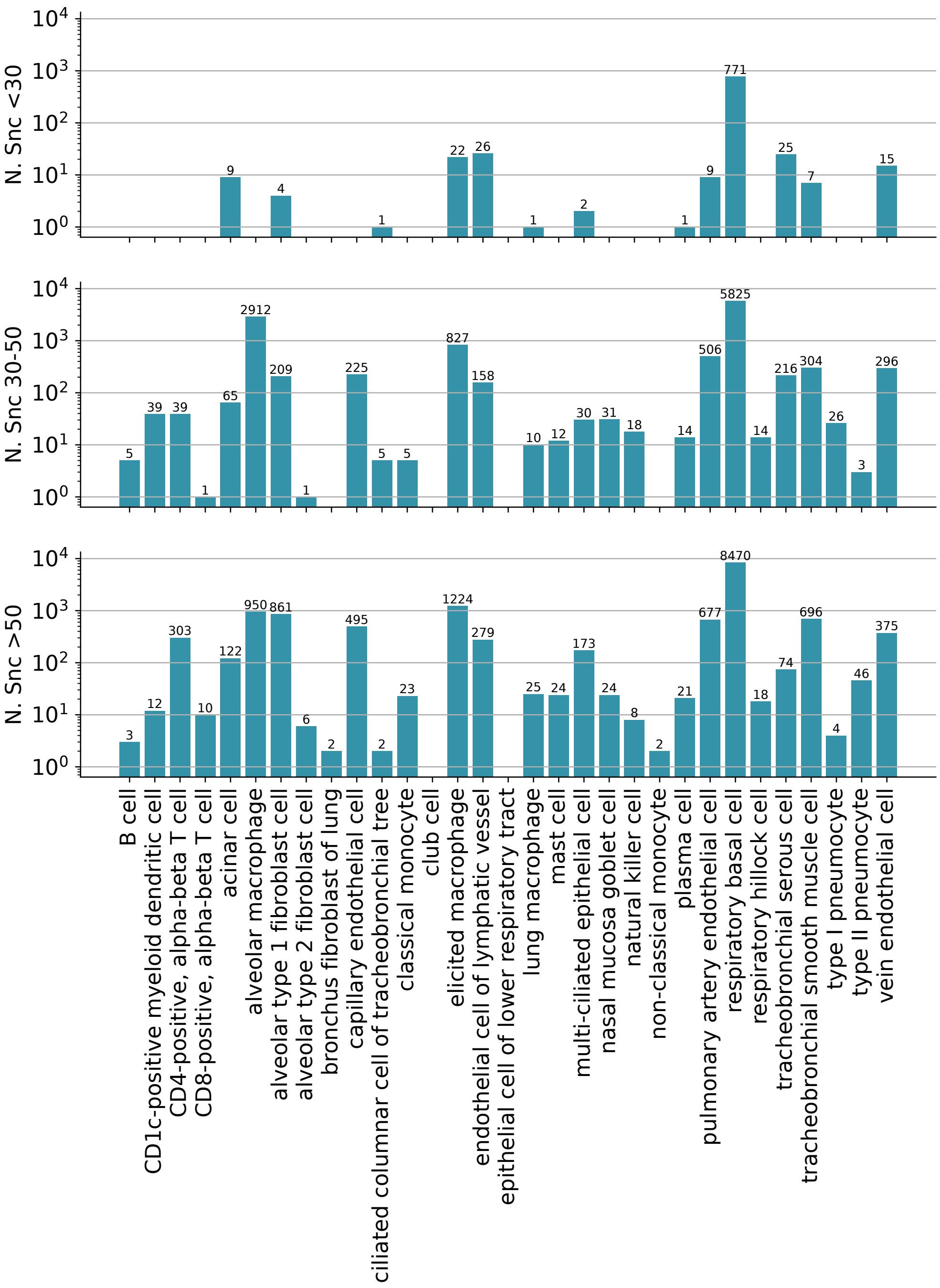

Figure 3: **Number of cells identified as senescent by the PU learner for each age group in the HLCA dataset.**

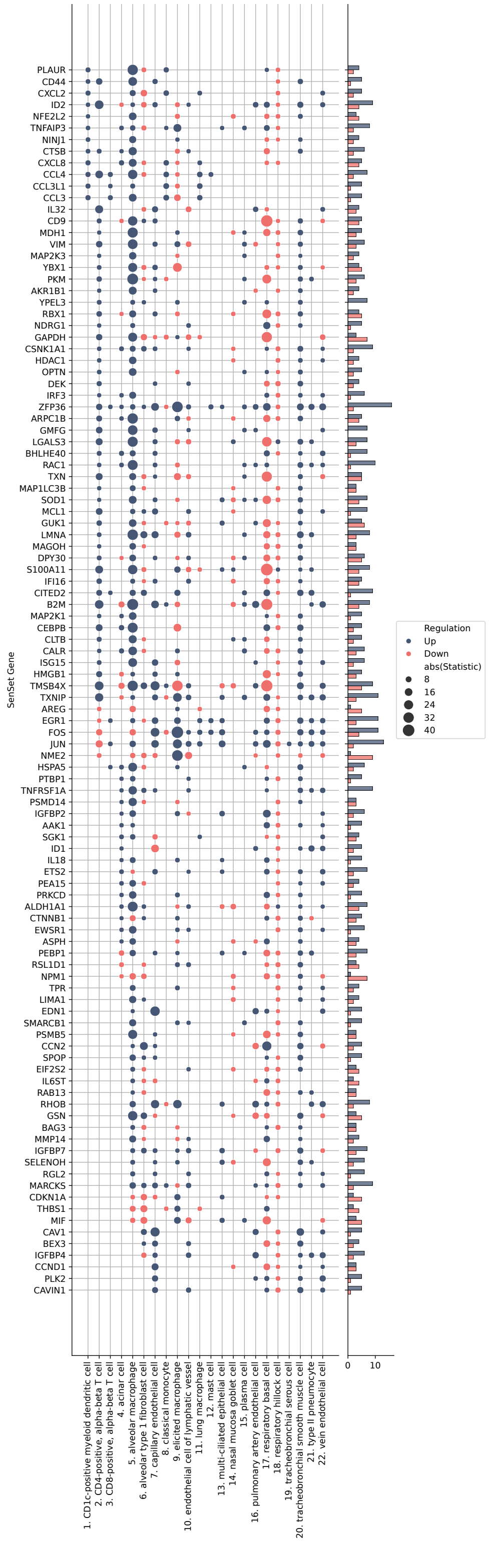

Figure 4: **All SenSet genes and their enrichment for 22 cell types in the HLCA dataset. The DE test was performed using a rank sum test.**

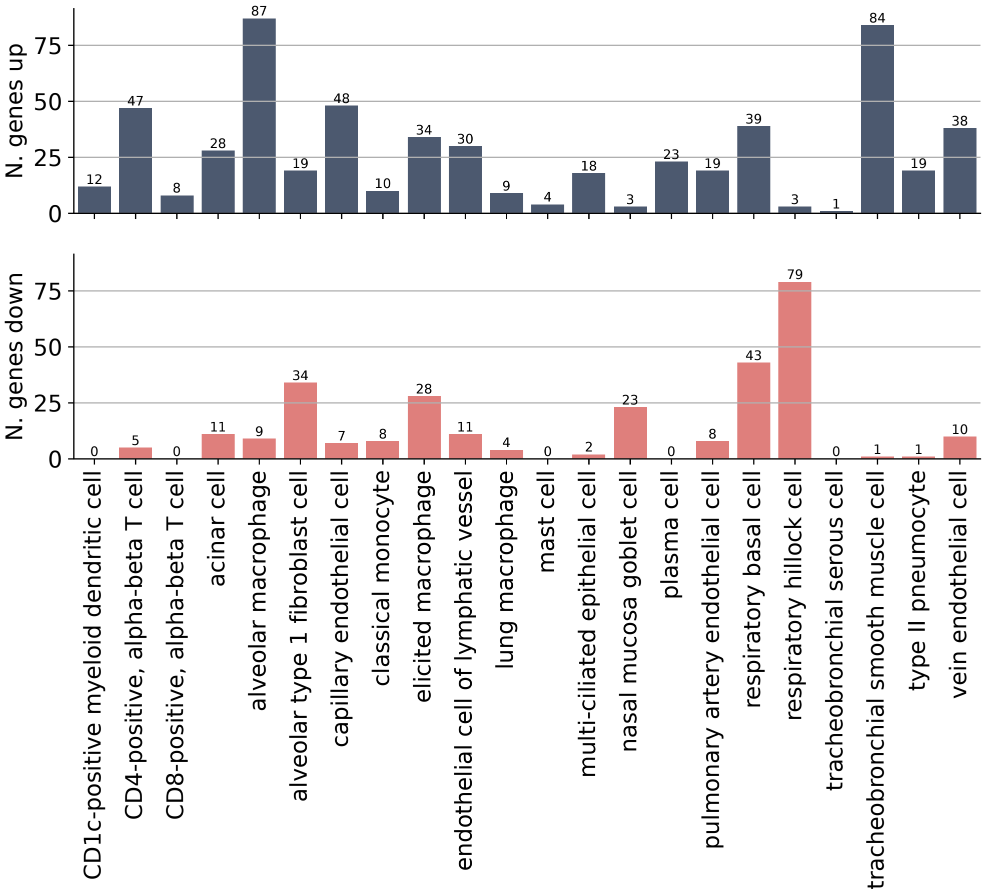

Figure 5: **Number of SenSet genes up and downregulated in the negative (senescent) class for each cell type in the oldest age group $\mathcal{A}$.**

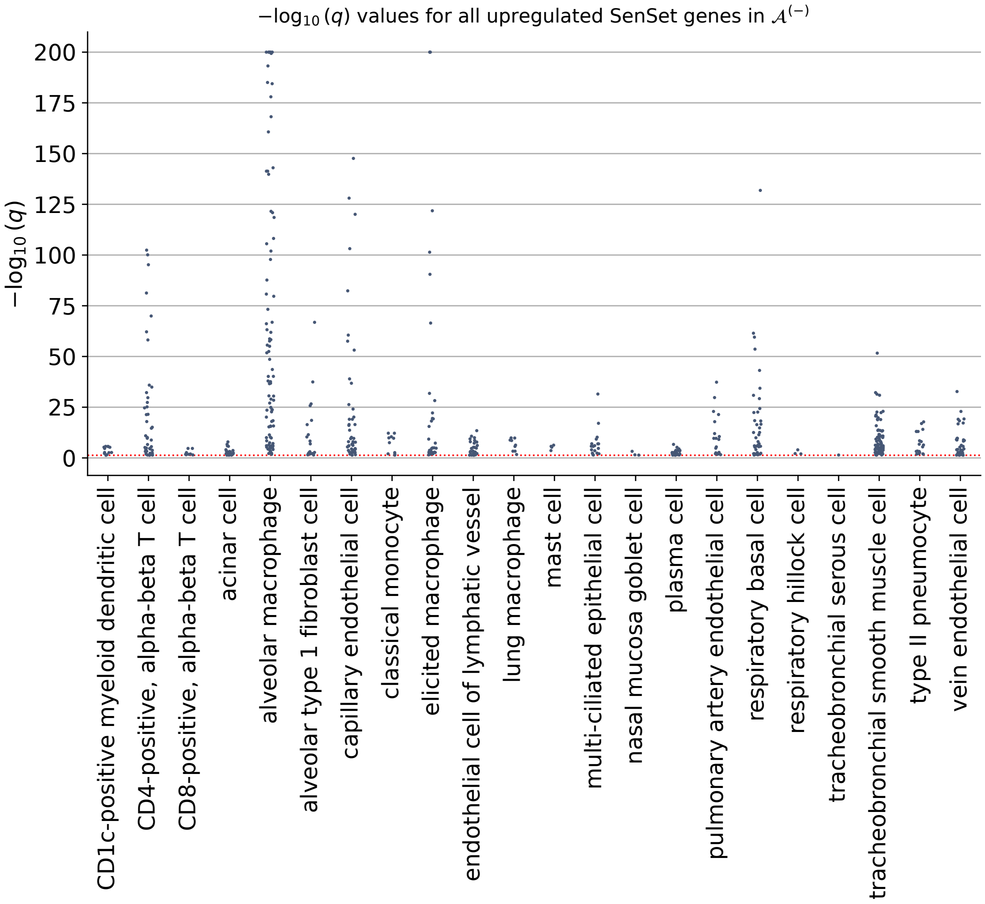

Figure 6: **Distribution of q-values for all SenSet genes upregulated in senescent cells across cell types in the oldest age group $\mathcal{A}$.**

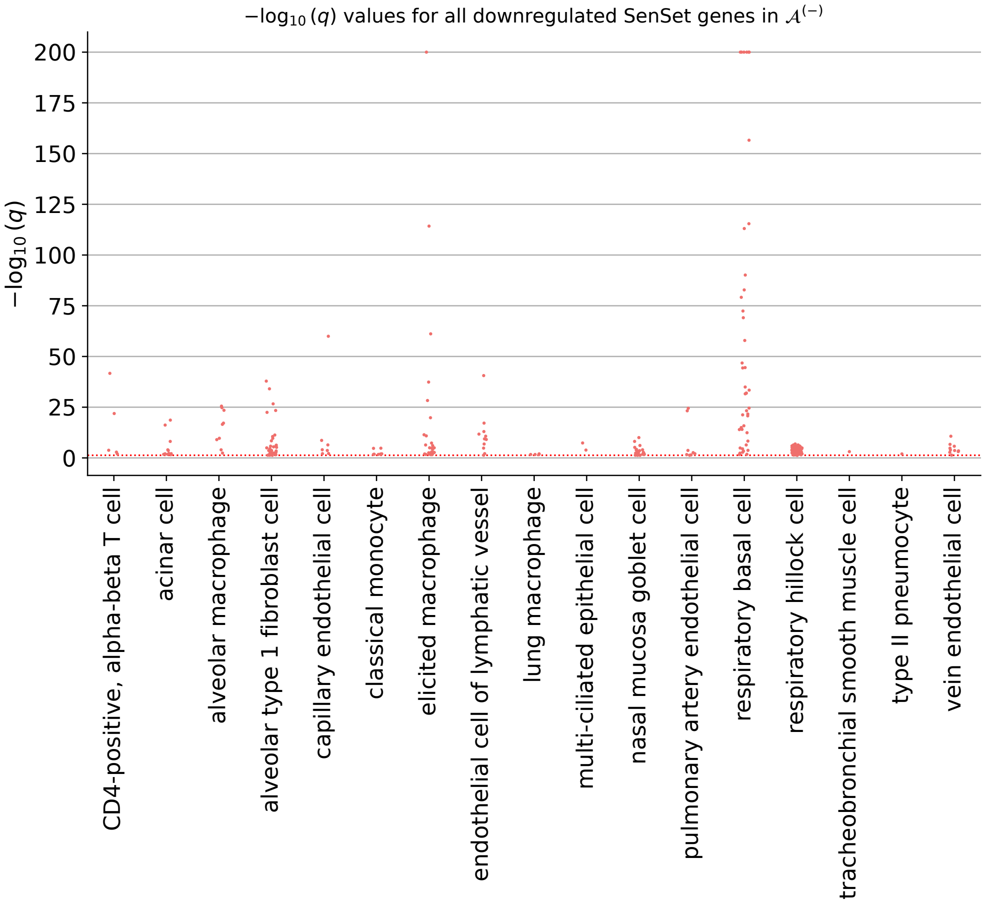

Figure 7: **Distribution of q-values for all SenSet genes downregulated in senescent cells across cell types in the oldest age group $\mathcal{A}$.**

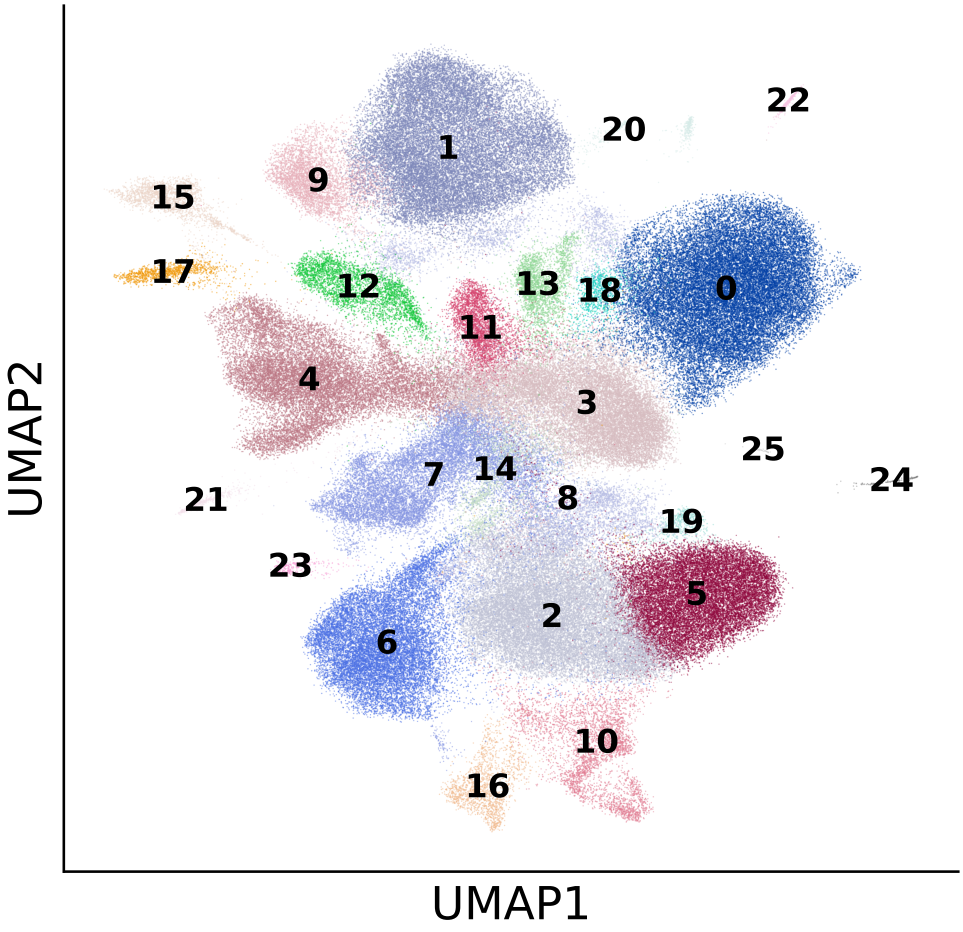

Figure 8: **All clusters identified in our PCLS data using the Leiden method.**

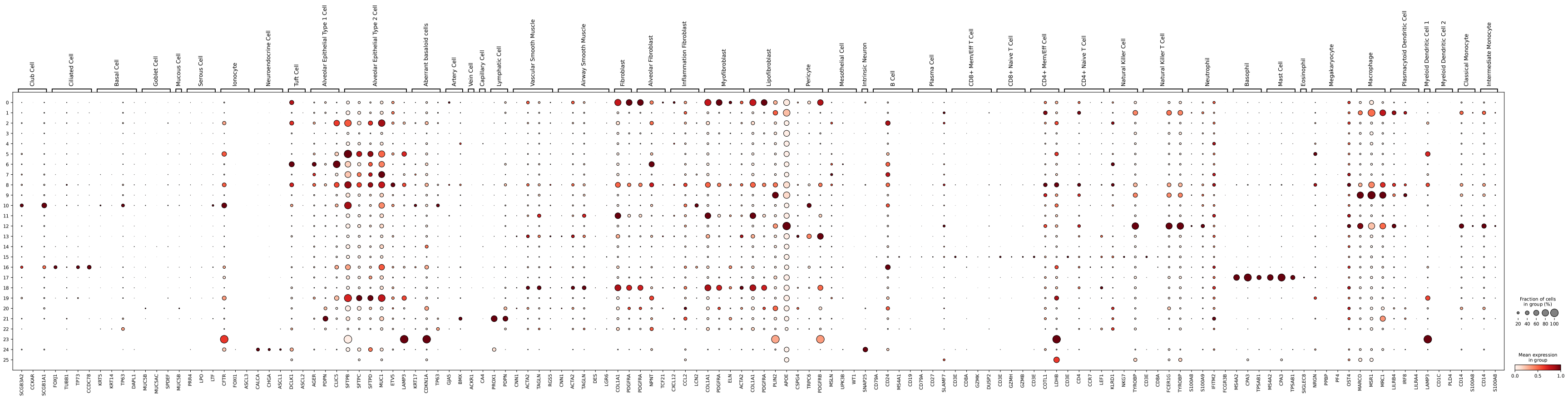

Figure 9: **Canonical markers used to identify cell types in the PCLS. These markers were obtained from Travaglini *et al.* [1] and were supplemented with additional markers for transient alveolar epithelial cells.**

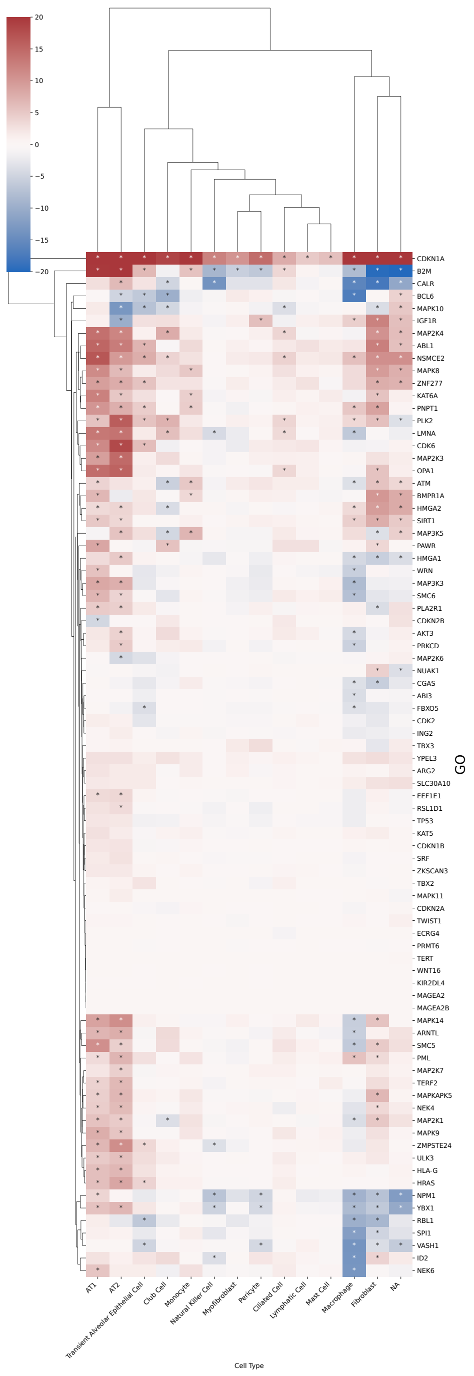

Figure 10: **Regulation of all marker genes in the GO set for each cell type in treated vs. control cells in our PCLS model.**

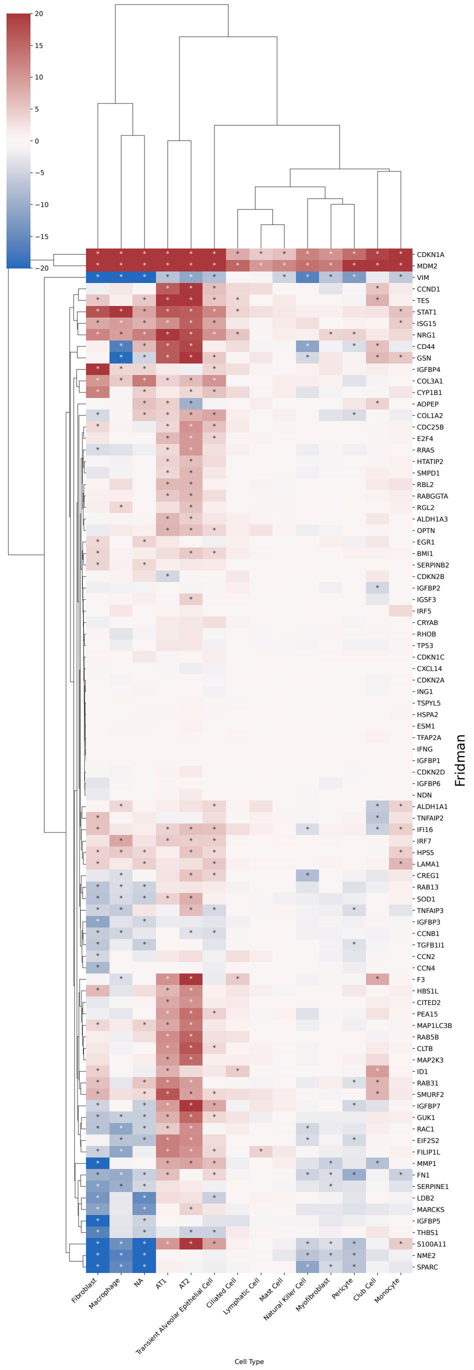

Figure 11: **Regulation of all marker genes in the Fridman set for each cell type in treated vs. control cells in our PCLS model.**

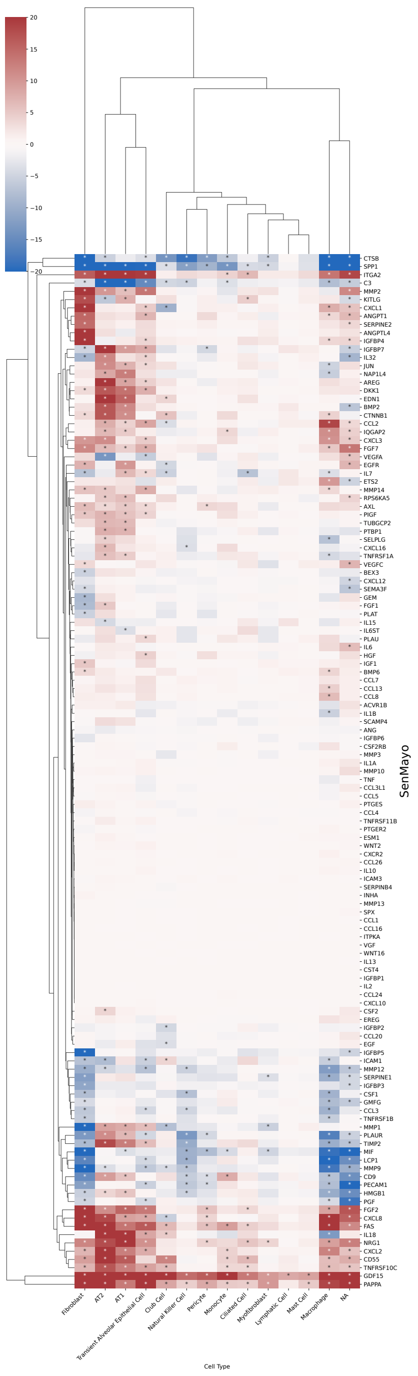

Figure 12: **Regulation of all marker genes in the SenMayo set for each cell type in treated vs. control cells in our PCLS model.**

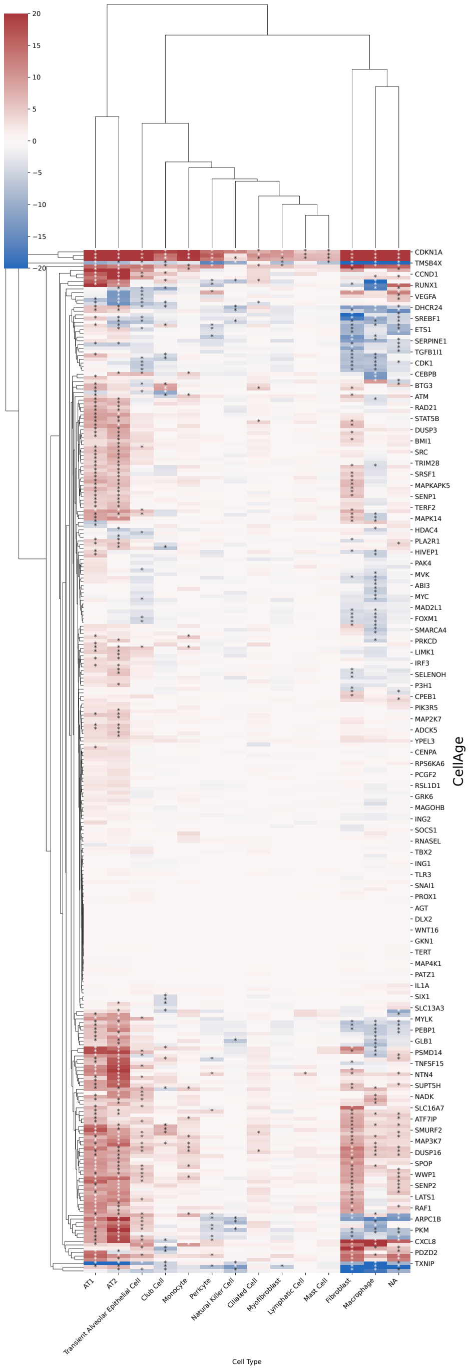

Figure 13: **Regulation of all marker genes in the CellAge set for each cell type in treated vs. control cells in our PCLS model.**

References

1. Travaglini, K. J. *et al.* A molecular cell atlas of the human lung from single-cell RNA sequencing. en. *Nature* **587,** 619–625 (Nov. 2020).
